# The Contralateral Repeated Bout Effect of Elbow Flexors Is Not Observed in Young Women Following Mild Muscle Damage from Eccentric Exercises

**DOI:** 10.3390/sports11030062

**Published:** 2023-03-09

**Authors:** Bailey A. Brown, Xin Ye, Ling Xin

**Affiliations:** 1 Snell Prosthetics and Orthotics, Little Rock, AR 72205, USA; 2 Department of Rehabilitation Sciences, University of Hartford, West Hartford, CT 06117, USA; 3 Exercise Science Program, Biology Department, Simmons University, Boston, MA 02115, USA

**Keywords:** eccentric exercise, muscle damage, elbow flexors, repeated bout effect, median frequency

## Abstract

Investigation of the contralateral repeated bout effect (CL-RBE) in women is scarce. Therefore, this study aimed at examining whether CL-RBE exists in women. Twelve healthy women (age: 20.9 ± 2.5 years) performed two bouts of maximal elbow flexor eccentric exercise (3 sets × 15 repetitions per bout) separated by 14 days, using the opposite arms. Surface Electromyography (EMG) was recorded during both exercise bouts. The isokinetic muscle strength (60°/s), muscle soreness, range of motion (ROM), limb girth, and blood creatine kinase activity were measured pre-exercise, and at 24 and 48 h post-exercise with the muscle strength being measured immediately post-exercise as well. Significant main effects of time were observed for muscle strength, muscle soreness and ROM (*p* < 0.05). There were no significant differences between bouts for all the measured variables including the EMG median frequency (*p* > 0.05). These results suggest that the CL-RBE of elbow flexors was not evident in young healthy women. This was because the mild muscle damage induced by the initial bout of exercise was either not enough to initiate the CL-RBE or the CL-RBE in women lasted shorter than two weeks. This study provides important information for future studies on CL-RBE in women.

## 1. Introduction

Eccentric (ECC) contraction is a type of muscle action during which a muscle is forced to be lengthened while it is trying to contract. Unaccustomed ECC exercise induces transient muscle damage [1]. Exercise-induced muscle damage (EIMD) is displayed by indirect markers including prolonged loss of muscle strength, decreased range of motion (ROM), delayed onset muscle soreness (DOMS), muscle swelling, and elevated blood level of creatine kinase (CK) activity [1,2,3], with muscle strength loss being considered the best EIMD marker [2,4].

It has been well documented that one bout of eccentric exercise provides a protective effect against muscle damage when the same exercise is conducted later by the same muscle [1,5,6,7], also known as the repeated bout effect (RBE). Since the second bout is performed by the same muscle, this is also referred to as the ipsilateral repeated bout effect (IL-RBE). The IL-RBE is characterized by a reduced magnitude of muscle strength loss, swelling, ROM loss, DOMS, and blood CK activity increase compared with the first bout [8,9,10]. The IL-RBE has been reported for arm, leg, and trunk muscles [5,7,10,11], as well as in both sexes [12,13]. It has been shown that the IL-RBE manifests itself one week after maximal eccentric exercises [14,15] and can last at least six months [16].

Increasing evidence has demonstrated that an initial bout of ECC exercise also produces protective adaptation in the contralateral limb, which is defined as the contralateral repeated bout effect (CL-RBE). Similar to the IL-RBE, the CL-RBE is manifested by attenuated muscle strength loss, swelling, DOMS, ROM reduction, and less increase in blood CK activity when the second bout of ECC exercise is conducted by the contralateral homologous muscle that was not involved in the initial bout of ECC exercise [8,9,17]. To date, the CL-RBE has been observed on elbow flexors [8,9,18,19], knee extensors [17], knee flexors [20], and tibialis anterior muscles [21]. The magnitude of the CL-RBE has been shown to be smaller than that of the IL-RBE in several studies [8,9,19,20], while a couple of studies found no significant difference between the magnitude of IL-RBE and CL-RBE [10,21]. Regarding the lasting duration of CL-RBE, Chen et al. [19] showed that the CL-RBE was evident when the second bout of eccentric exercise was performed by the contralateral arm at one day, one week, or four weeks, but not within 12 h or eight weeks, after the first eccentric exercise. The same research group [20] also reported that the knee flexor CL-RBE was manifested when the two eccentric exercise bouts were separated by one or seven days, but it disappeared when the second eccentric exercise bout was performed by the other leg four weeks after the first exercise. According to the research work by Chen and colleagues [19,20] as well as other researchers [8,9,17], the CL-RBE appears to last from one day to four weeks, depending on the eccentric exercise protocol and the muscle groups involved.

Several mechanisms have been proposed to explain the RBE. The IL-RBE appears to be the combined consequence of neural, mechanical, and cellular adaptations [6,22]. The CL-RBE may be accomplished via neural adaptation [9,21] or attenuation of pro-inflammatory responses [17]. Neural adaptations are the most likely explanation for the CL-RBE because no direct stress/damage was exerted on the contralateral muscles during the first eccentric exercise. Indeed, using surface electromyography (EMG), Starbuck and Eston [9] observed a significant decrease in the EMG median frequency (MF) during the second bout of eccentric exercise by the contralateral arm. MF is a variable recommended for the study of muscle fatigue and damage [23]. A decrease in MF indicates an increase in the recruitment of slow-twitch motor units. It is known that eccentric contractions preferentially recruit fast-twitch motor units [24]. In addition, fast-twitch muscle fibers are more susceptible to eccentric exercise-induced muscle damage [25]. Therefore, the shift from greater recruitment of fast-twitch motor units to slow-twitch motor units might decrease the stress to the vulnerable fast-twitch muscle fibers; thus, this could provide a potential mechanism for the CL-RBE. The involvement of neural adaptations in the CL-RBE was also supported by another study [21] which showed that the decrease in the nociceptive withdrawal reflex threshold (NWRT) during the first eccentric exercise was abolished after the second exercise by the contralateral TA, indicating spinal facilitation during the CL-RBE. Overall, the data on the CL-RBE mechanisms are still very limited and need further investigation.

To the best of our knowledge, all the previous studies investigating the CL-RBE only recruited males [10,17,19,20,21,26] or a mixture of both sexes [27,28]. This is somewhat surprising, given the practical application of the CL-RBE in clinical and rehabilitation settings [17]. For example, when one limb is immobilized due to an injury or a disease, eccentric-exercise training the contralateral healthy limb muscles before training the immobilized limb muscles can minimize the potential muscle damage that could occur to the immobilized limb during the rehabilitative process. Since a training program should ideally be a scientific-based guide, it is critical to conduct research to extensively characterize the existence and magnitude of the CL-RBE in different populations. The lack of information about the CL-RBE in women will thus impede its application. In addition, there is discrepancy in the literature regarding muscle damage responses and adaptations of men and women during/after eccentric exercises. Lee et al. [29] reported no sex differences in muscle recovery after 150 eccentric contractions of the knee extensor muscles. Similarly, O’Connor et al. [30] demonstrated that men and women had similar perceived exertion responses to increased intensity of elbow extension exercises (80%, 100%, or 120% of maximal voluntary concentric strength). However, Power and colleagues [31] showed that women had a greater loss of power than men at higher force load (60% maximum voluntary isometric contraction), along with a slower recovery following 150 eccentric actions of the ankle dorsiflexors. In contrast, a recent study by West et al. [32] demonstrated that women experienced a faster recovery of isometric strength and muscle soreness following a bout of knee-extension eccentric exercise-induced muscle damage. These findings suggested that data from men regarding muscle damage response to eccentric exercise might not be applicable for women.

Therefore, the purpose of this study was to investigate whether the CL-RBE of elbow flexors exists in women. Since a few studies [7,13,33] demonstrated the existence of the IL-RBE in women, similar to that seen in men, and we are not aware of any reported sex-related differences in the CL-RBE, we hypothesized that this phenomenon (CL-RBE) can happen to women as well. To test this hypothesis, young women were recruited, and each completed maximal eccentric contractions using the opposite arms in two bouts separated by 14 days. Surface EMG was recorded during both exercise bouts from the biceps brachii muscle. The isokinetic muscle strength was measured pre-exercise, immediately post-exercise, and at 24 and 48 h post-exercise. Muscle soreness, ROM, limb girth, and blood CK activity were measured pre-exercise, and at 24 and 48 h post-exercise. We hypothesized that the CL-RBE of elbow flexors exists in women and that this would be characterized by an attenuated decrease in muscle strength and ROM, less DOMS, less increase in limb girth and blood CK during the second eccentric exercise by the contralateral arm. We also hypothesized that there would be a reduction in MF during the second bout of eccentric exercise by the opposite arm.

## 2. Materials and Methods

### 2.1. Subjects

A priori power analysis was conducted to estimate the required number of subjects for this study using G*Power (G*Power 3.1.9. 4, Heinrich-Heine-University Düsseldorf, Düsseldorf, Germany). On the basis of an expected small effect size (Cohen’s d = 0.25), an α level of 0.05, and a power (1-β) of 0.8 [34], it was estimated that 11 subjects were necessary. We originally recruited 14 subjects; however, two of them dropped out. Therefore, 12 untrained, healthy young (18–30 years) women volunteered and completed all the visits. Their mean ± SD age, height, and body mass were 20.9 ± 2.5 years, 162.9 ± 7.5 cm, and 66.2 ± 10.4 kg, respectively. Subjects were recruited from the University of Mississippi via recruitment fliers placed in the Student Union and Turner Center, or mass emails to the enrolled female students at University of Mississippi. The subjects had not participated in resistance training of arms for at least six months prior to the study. All the subjects had no prior history of surgeries or injuries to the arm, neck, or wrist. They completed a Physical Activity Readiness Questionnaire (PAR-Q) [35] and a medical history questionnaire to ensure that they were able to perform the exercise and measurements safely. No subjects had been diagnosed with cardiovascular, pulmonary, metabolic, or any other chronic diseases, and had no routine use of any medications except birth control pills. The subjects were asked to refrain from using oral and topical analgesics, heat or cold treatment, physical therapy, massage, or any other muscle treatment regimen during the course of the study. All the subjects were informed of the study protocol and signed the informed consent documents that had been approved by the Institutional Review Board of the University of Mississippi (protocol #:18-058).

### 2.2. Study Design

**Figure 1:**
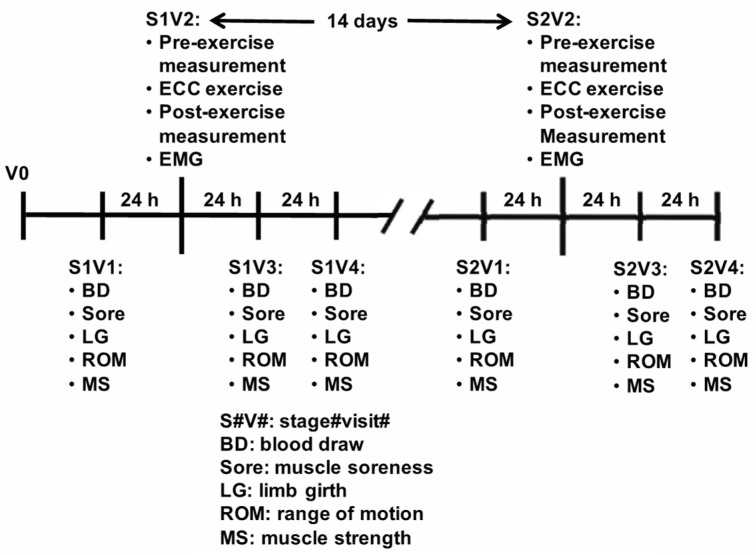
Study layout.

All the subjects completed nine separate visits as illustrated in Figure 1. The pre-test screening was conducted over the phone. After phone screening, the pre-qualified subjects were scheduled for an interview visit (V0). During V0, the subject reviewed and signed the informed consent form and completed the brief medical history and PAR-Q forms. In addition, the arm to be tested (exercised) in the initial bout of eccentric exercise was determined by alternating from dominant to non-dominant arm as the subjects were enrolled. Therefore, there were equal numbers of subjects who used their dominant or non-dominant arm in the first bout of eccentric exercise. The eligible subject then completed eight visits, which were divided into two stages (Stage 1: Visits 1–4, and Stage 2: Visits 1–4). During the Stage 1 Visit 1 (S1V1), the subjects’ height and weight were measured, and the dates of the first day of their last menstrual cycle were recorded. S1V1 consisted of baseline measurements of muscle soreness, ROM, limb girth of the upper arm, and isokinetic muscle strength. Blood was drawn from the antecubital vein before the isokinetic muscle strength measurement, but after the assessment of muscle soreness, ROM, and limb girth. Twenty-four hours after the completion of S1V1, each subject returned for her Stage 1 Visit 2 (S1V2). At the beginning of S1V2, muscle soreness, ROM, limb girth, and isokinetic muscle strength of the exercise-assigned arm were measured. The subjects then completed 45 maximal eccentric actions of the assigned bicep muscles on an isokinetic dynamometer. Immediately after the eccentric exercise, the isokinetic muscle strength of the arm was measured. On each day of the following two days (S1V3 and S1V4) after S1V2, the subjects had blood drawn and completed the limb girth, muscle soreness, ROM, and strength assessment of the exercised arm. Stage 2 Visit 1 (S2V1) was 13 days after S1V2. Stage 2 Visits 1–4 were the same as Stage 1 Visits 1–4, except that the opposite arm was exercised and tested.

### 2.3. Eccentric Exercise

Eccentric exercise was conducted on an isokinetic dynamometer (Biodex System 3, Biodex Medical Systems, Inc., Shirley, NY, USA). The subjects were seated so that their backs were straight and leaning against the back of the seat of the dynamometer, and straps were used to secure their shoulders and waist. The arm rest was raised so that their arm was comfortably resting at a 45° angle. The subject’s upper arm was supported by the Biodex attachment. The tested/exercised hand was positioned in a supinated position and the subjects could comfortably flex and extend their arm. The handlebar was lined up so that the subject’s lateral humeral epicondyle was aligned with the axis of rotation of the dynamometer. The settings of the dynamometer were recorded to ensure that every visit had the same position as the initial visit. The subjects performed 3 sets of 15 repetitions of maximal eccentric contractions using the elbow flexor at a speed of 30°/s with 10 s of rest in between each repetition and 3 min of rest between each set. We chose this exercise protocol because it effectively resulted in a significant amount of muscle damage in a previous CL-RBE study in men [8]. A similar eccentric exercise protocol was also used in two other previous CL-RBE studies in men, except that they utilized 30 (5 sets × 6 repetitions) [19] and 60 (6 sets × 10 repetitions) [9] as the total number of the maximal eccentric contractions, respectively. We chose the protocol of 45 eccentric contractions because we were not sure if 30 eccentric contractions would be too few or 60 repetitions would be too many to induce a significant but not too strong damaging stimulus in women. The starting position was 30° from the subject’s arm at full flexion and ended at full extension (0°). The subject was verbally encouraged to pull maximally during each eccentric contraction. At the end of each eccentric contraction, the subject’s arm was moved back to the starting position by the investigator. The work during each set was measured, and the total work accomplished during each bout of eccentric exercise was calculated by adding all the work done during each of the three sets, which was measured by and exported from the Biodex dynamometer system.

### 2.4. Electromyography (EMG)

During each bout of the eccentric exercise, surface EMG signals were recorded through a wireless EMG sensor (Trigno™ EMG Sensor, Delsys, Inc., Natick, MA, USA). The EMG electrode was attached over the biceps brachii muscle belly according to the sensor location recommendations from SENIAM [36]. Prior to any electrode placements, the skin site was shaved with a razor and cleaned with rubbing alcohol. In addition, medical tape was used to firmly fix the electrode on the skin. The analog bipolar EMG signals were collected and amplified (gain = 1000) with a Trigno™ Wireless System (Delsys, Inc., Natick, MA, USA) and filtered with high- and low-pass filters set at 20 and 450 Hz, respectively. The filtered signals were then digitized at a sampling rate of 1926 Hz.

For the calculation of EMG median frequency (MF), the EMG signal was first filtered with a Hamming window, and then the Discrete Fourier Transform (DFT) algorithm was used to derive the EMG signal into the power spectrum. Lastly, the MF of the spectrum was calculated based on the equation described in the paper by Kwatny et al. [37]. For each of the eccentric muscle contractions, the mid 1 s portion of the entire 3 s was selected for analysis.

### 2.5. Isokinetic Muscle Strength

In 2018, Chen et al. [38] demonstrated that two maximal voluntary isometric contractions of one arm attenuated the muscle damage induced by maximal eccentric exercise performed by the contralateral arm. To avoid the potential protective effect of maximal isometric contractions, the current study measured isokinetic muscle strength instead of isometric strength as an indirect market of EIMD. Isokinetic muscle strength assessment was conducted on the Biodex System 3 isokinetic dynamometer. The same positioning of the subject on the Biodex dynamometer for the eccentric exercise was used for the muscle strength measurements. The subjects did three maximal biceps curls at the angular velocity of 60°/s throughout the full range of elbow motion. The peak torque was the highest torque value the subject produced during the three trials.

### 2.6. Muscle Soreness

Each subject was asked to do two bicep curls with either a 0.45 kg or 0.9 kg dumbbell. The subject used a 0.45 kg weight if her body mass was under 59 kg, and a 0.9 kg dumbbell was used if she weighed over 59 kg. After the two curls, she was asked to mark her peak soreness level of the tested arm on a 100 mm visual analog scale (VAS), with “no pain” on the left end (0 mm) and “unbearable soreness” on the right end (100 mm). The distance from the left end to the mark was measured and recorded as the soreness level.

### 2.7. Range of Motion (ROM)

A goniometer was used to measure the ROM of the arm being tested. The subjects were asked to keep their arm down by their side and extend it as far as they could comfortably. They were then asked to flex the arm maximally with their palm facing their shoulder. The maximal degree of flexion and extension were recorded. The full range of motion was determined by subtracting the maximal flexed joint angle from the maximal extended joint angle.

### 2.8. Limb Girth

Each subject was asked to relax their arms at their side, and the length from the acromion to the lateral epicondyle of the tested arm was measured using a tape measure. The mark was placed at 2/3 of the length from the acromion to the lateral epicondyle. The circumference of the upper arm surrounding the mark was taken three times. The circumferences were then averaged and recorded. The subject was instructed not to wash away the mark on the tested arm so that the same location was used for the limb girth measurement during the subsequent visits.

### 2.9. Blood CK Activity Assay

Blood samples were collected from the antecubital vein and were then spun in the centrifuge for 15 min at 3000 rpm at 4 °C to separate the plasma from the other contents of the blood. The plasma samples were stored in the −80 °C freezer until ready to transport. After a subject’s blood samples from all visits were collected, the plasma samples were packed up and shipped to North Mississippi Medical Center (NMMC) where the CK activity level was measured using a VITROS VITROS^®^ XT 7600 Integrated System (Ortho-Clinical Diagnostics, Inc., Rochester, NY, USA).

### 2.10. Statistical Analyses

Isokinetic muscle strength, muscle soreness, ROM, limb girth, and CK data were analyzed using two-way repeated measures analysis of variance (ANOVA) to determine the main effects of time (exercise), bout, and their interaction terms. A two-way (set-bout) repeated measures ANOVA was utilized to analyze the EMG MF data. A paired t-test was used to compare the total work in eccentric exercise Bout 1 and Bout 2. When appropriate, Bonferroni’s post hoc analysis was performed. Eta-squared values (η^2^) were calculated as measures of effect size. A significance level was set at *p* < 0.05. All data analyses were conducted using SPSS statistical software (Version 22, SPSS Inc., Chicago, IL, USA).

## 3. Results

### 3.1. Baseline Measurements

Table 1 displays the baseline and pre-exercise values of muscle strength, ROM, and limb girth. No significant differences between the two bouts were observed for any of these variables.

### 3.2. Eccentric Exercise

The exercising ROM was 107.7 ± 9.1° and 104.45 ± 7.3° for Bout 1 and Bout 2, respectively. The exercising ROM difference between Bout 1 and Bout 2 was not statistically significant (*p* = 0.20). Similarly, no significant difference (*p* = 0.69) was observed between the total work completed in the eccentric exercise Bout 1 (469.4 ± 165.5 J) and Bout 2 (453.4 ± 187.9 J). These results indicate that the subjects provided similar and consistent efforts during both eccentric exercise bouts, and this thus minimizes the potential confounding effects from the difference in both exercise bouts.

### 3.3. Electromyography (EMG)

**Figure 2:**
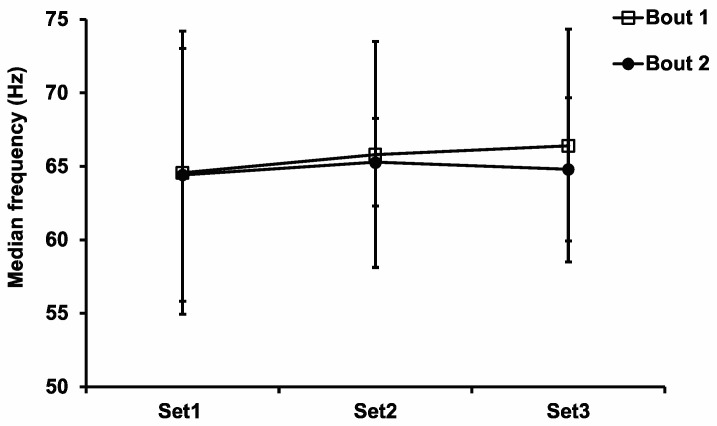
Median frequency (MF) of the 3 sets during both eccentric exercise bouts. Values are mean ± SD; *n* = 8 for each time point.

Median frequency (MF) was recorded and processed from the raw EMG data. Four subjects’ EMG data were omitted due to EMG equipment errors during testing. As a result, only 8 subjects’ EMG data were used for the final analyses. Statistical analysis of the MF data revealed no significant difference for the main effects of the set (F_2,14_ = 0.38, *p* = 0.69, η^2^ = 0.005) or bout (F_1,7_ = 0.20, *p* = 0.67, η^2^ = 0.003) (Figure 2), suggesting that the MF did not change significantly during Bout 2 compared with Bout 1.

### 3.4. Isokinetic Muscle Strength

**Figure 3:**
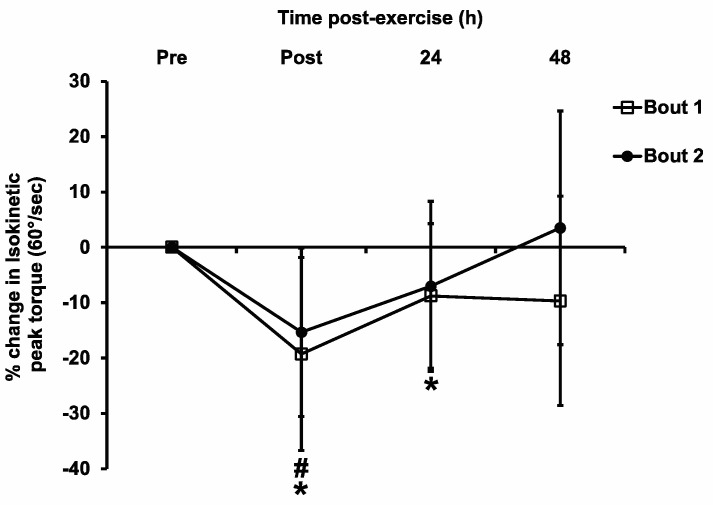
Isokinetic contraction peak torque pre-exercise (Pre), immediately post-exercise (Post), and 24 h and 48 h post-exercise following the two bouts of eccentric exercise. Values are mean ± SD; *n* = 11 for each time point. * Significant difference compared with pre-exercise in Bout 1. # Significant difference compared with pre-exercise in Bout 2.

Figure 3 shows the percent changes in isokinetic peak torque at 60°/s at different time points after the two bouts of eccentric exercise. There was a technical problem with the Biodex dynamometer when we measured the muscle strength for one subject. Therefore, the isokinetic peak torque data were collected from 11 subjects. There were significant main effects of time (F_3,30_ = 7.69, *p* = 0.001, η^2^ = 0.053). The maximal isokinetic torque loss was observed immediately post-exercise for both exercise bouts, decreasing by 19.3 ± 17.4% (*p* < 0.01) and 15.3 ± 15.2% (*p* < 0.01) in Bout 1 and Bout 2, respectively. The isokinetic peak torque returned to pre-exercise levels at 48 h post-exercise in Bout 1 and 24 h post-exercise in Bout 2, respectively, which seemed to suggest a faster muscle strength recovery after Bout 2. However, there were no significant bout (F_1,10_ = 0.49, *p* = 0.50, η^2^ = 0.003) or interaction (F_3,30_ = 1.08, *p* = 0.37, η^2^ = 0.008) effects.

### 3.5. Muscle Soreness

**Figure 4:**
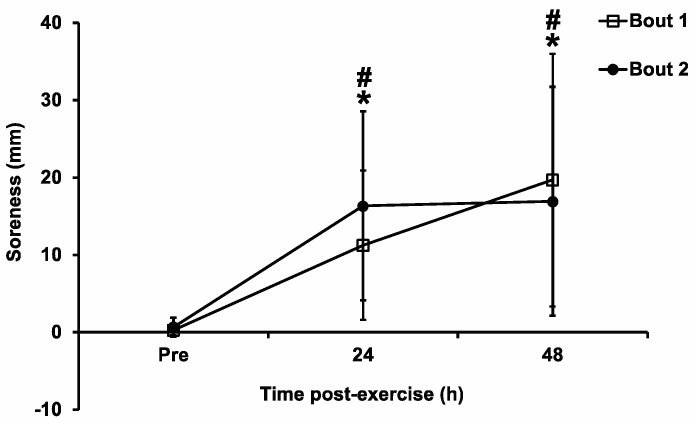
Muscle soreness pre-exercise (Pre), and 24 h and 48 h post-exercise following the two bouts of eccentric exercise. Values are mean ± SD; *n* = 12 for each time point. * Significant difference compared with pre-exercise in Bout 1. # Significant difference compared with pre-exercise in Bout 2.

There were significant main effects of time (F_2,22_ = 15.86, *p* < 0.001, η^2^ = 0.334) for muscle soreness. Compared with the baseline level, muscle soreness was significantly (*p* < 0.05) increased at both 24 h and 48 h post-exercise within each bout, indicating that the exercise protocol was successful in inducing muscle damage. However, there were no significant bout (F_1,11_ = 0.23, *p* = 0.64, η^2^ = 0.001) or interaction (F_2,22_ = 2.27, *p* = 0.13, η^2^ = 0.015) effects (Figure 4).

### 3.6. Range of Motion

**Figure 5:**
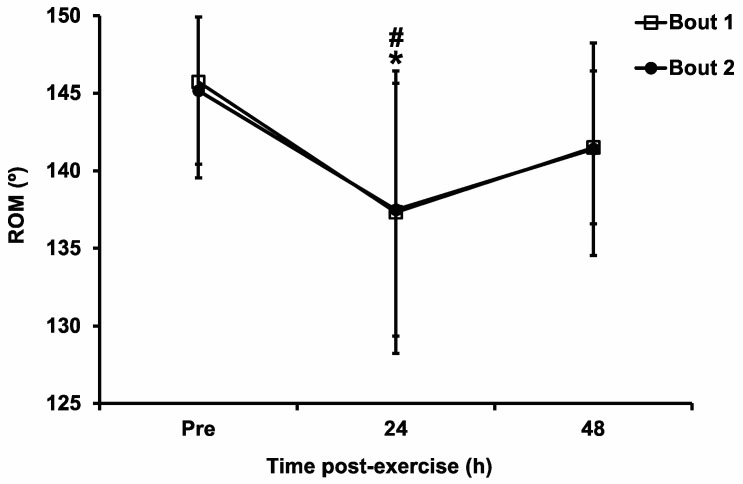
Range of motion (ROM) pre-exercise (Pre), immediately post-exercise (Post), and 24 h and 48 h post-exercise following the two bouts of eccentric exercise. Values are mean ± SD; *n* = 12 for each time point. * Significant difference compared with pre-exercise in Bout 1. # Significant difference compared with pre-exercise in Bout 2.

Compared with the baseline value, ROM was significantly decreased at 24 h after exercise in Bout 1 (*p* = 0.03) and Bout 2 (*p* = 0.001) (Figure 5). However, there were no significant bout (F_1,11_ = 0.01, *p* = 0.92, η^2^ = 0.0001) or interaction (F_2,22_ = 0.03, *p* = 0.97, η^2^ = 0.0004) effects.

### 3.7. Limb Girth

The upper limb girth did not differ significantly over time (F_2,22_ = 0.15, *p* = 0.86, η^2^ = 0.0001) or between the two bouts ((F_1,11_ = 0.06, *p* = 0.82, η^2^ = 4.2 × 10^−5^) of exercise. Neither was any significant difference (F_1,10_ = 0.01, *p* = 0.99, η^2^ = 7.9 × 10^−6^) identified in the interaction of time and bout.

### 3.8. Blood CK Activity

**Figure 6:**
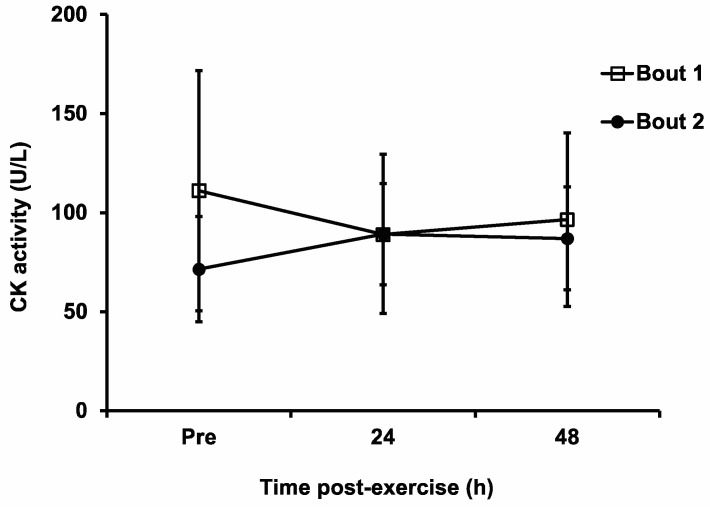
Plasma creatine kinase (CK) activity pre-exercise (Pre), and 24 h and 48 h post-exercise following the two bouts of eccentric exercise. Values are mean ± SD; *n* = 11 for each time point.

We could not obtain blood samples from one subject due to the small size of her antecubital vein. Therefore, the CK activity data were collected from the blood samples of 11 subjects. There were no significant main effects of bout (F_1,10_ = 4.04, *p* = 0.07, η^2^ = 0.043), time (F_2,20_ = 0.04, *p* = 0.96, η^2^ = 8 × 10^−4^) or their interaction (F_2,20_ = 2.51, *p* = 0.11, η^2^ = 0.047) in the plasma CK activity level (Figure 6).

## 4. Discussion

The main objective of this study was to examine whether the CL-RBE of elbow flexors exists in women. We observed significant main effects of time for muscle strength loss, DOMS, and ROM, suggesting that the eccentric exercise protocol used in this study successfully induced muscle damage. However, no significant main effects of bout or time–bout interactions were identified for all the measured variables. Therefore, the overall results of this study did not indicate the existence of a CL-RBE of the elbow flexors in young women.

In contrast to our hypothesis, the data of the current study did not support the presence of a CL-RBE in untrained young women. There were no significant differences in the commonly measured EIMD markers (muscle strength, muscle soreness, ROM, limb girth, blood CK activity, etc.) between the two bouts of eccentric exercise using the opposite arms. Although the magnitude of muscle strength loss seemed to be attenuated in the Bout 2 exercise, the difference between the bouts did not reach statistically significant levels. There are two main possible reasons why the CL-RBE was not evident in this study. First, the eccentric exercise protocol used was probably not damaging enough to the biceps brachii in the female subjects of this study, so it could not result in muscle adaptation in the contralateral arm. The magnitude of the muscle strength loss in the current study was smaller than those in previous studies [8,19]. Chen et al. [19] used a similar exercise protocol in men where the subjects performed 30 repetitions of eccentric elbow flexor exercises, and they found a ~40% decrease in muscle strength in the initial bout. Howatson and van Someren [8] used the same exercise protocol as in this study in which the male subjects performed two bouts of 45 maximal eccentric contractions using opposite arms, and they reported a ~25% muscle strength loss in the first bout. In the current study, the loss of muscle strength in the Bout 1 exercise was less than 20%, which was a smaller loss than the aforementioned two studies (~40% and ~25% strength loss). Based on the maximal voluntary contraction (MVC) force loss after damaging exercise, EIMD has been categorized as mild (MVC loss < 20%), moderate (MVC loss = 20–50%), or severe (MVC loss > 50%) (see the detailed definitions in the review article by Stožer et al. [39]). According to this categorization, the eccentric exercise protocol in our study induced mild muscle damage with a less than 20% MVC reduction. In contrast, the similar eccentric exercise protocols resulted in moderate muscle damage in the studies by Chen et al. [19] and Howatson and Van Someren [8]. Furthermore, we recognized that the muscle force reduction lasted much longer (at least 48 h) in previous studies [8,19] than in our study (only 24 h in Bout 1 and <24 h in Bout 2). The different magnitude and duration of muscle strength loss could be due to the sex difference of the recruited subjects between the current study and those two previous studies. Indeed, female skeletal muscles have displayed less damage following damaging stimuli such as strenuous exercise in both human and animal models [40,41]. Estrogen has been proposed to have a protective role in the inflammatory response following muscle damage [40]. Second, the initial bout of eccentric exercise might have generated enough stimulus to induce CL-RBE in women, but the CL-RBE lasted less than 2 weeks in women so it was not detected in the current study. We used 2 weeks as the time interval between the two exercise bouts based on previous studies [8,9,10], but the CL-RBE in women probably already subsided and become undetectable prior to 2 weeks. This is possible since Chen et al. [19] reported that magnitude of the CL-RBE of elbow flexors in men decreased with increasing time interval when the second eccentric exercise bout was performed at 1 day, 1 week, or 4 weeks after the first exercise bout. Compared to the peak isokinetic muscle strength loss in this study (~40%), our study showed a smaller isokinetic muscle strength loss (~20%) after Bout 1 exercise. It is reasonable to assume that the CL-RBE in our study, if it existed, would have subsided, and been abolished within a shorter time frame (such as two weeks), and thus we might have missed the time point to detect the CL-RBE. Due to the paucity of data about CL-RBE in women, we cannot find evidence in the literature to support our speculation. Therefore, future studies on CL-RBE in women need to adopt shorter time intervals between exercise bouts, such as 1 week or even shorter, so that the CL-RBE in women might be more profound and become detectable. Regardless of the possible mechanisms, the CL-RBE may not have been observed in this study because the initial eccentric exercise did not induce enough damaging stimulus to enact the CL-RBE or we might have missed the time interval when the CL-RBE in women was detectable.

There was no significant change in the limb girth throughout either exercise bout in this study. The limb girth may not have significantly changed because there was not enough inflammation to cause a detectable difference in the circumference of the upper arm. Chen et al. [19] found that there was a significant increase in limb girth after both exercise bouts, and there was a significant bout main effect. They used a similar protocol, which seemed to induce more muscle damage in men than in the women in the current study, based on the higher muscle strength loss (~40% loss) in their study.

Unaccustomed ECC exercise tends to have a greater reliance on, and therefore greater disruption to, fast-twitch motor units [9]. Surface EMG has been used to record motor unit activation in the muscle. It has been suggested that a higher MF indicates that there is a higher recruitment of fast-twitch muscle fibers, and a lower MF suggests that there is a higher recruitment of slow-twitch muscle fibers. An increased use of slow-twitch muscle fibers is known to be associated with the IL-RBE [22]. Starbuck and Eston [9] observed an increased recruitment of slow-twitch motor units in the contralateral arm during the second bout of eccentric exercise, and they concluded that the CL-RBE they observed was due to neural adaptation, specifically due to higher dependence on slow-twitch motor unit recruitment in the second bout of exercise. In the current study, there was no significant difference in the EMG MF between the first and second bouts. As discussed for the muscle strength data, the MF difference was not statistically significant, probably due to the insufficient damage stimulus generated by the first bout of eccentric exercise or because the CL-RBE had already subsided by the time we performed the measurements at 2 weeks. This speculation is reasonable because the study by Starbuck and Eston [9] demonstrated a reduction in EMG MF in Bout 2 vs. Bout 1. In that study, the strength decrement after the first bout was 25%, which is greater than our value.

Blood CK activity has been the most frequently used blood biomarker for EIMD [1]. The use of any blood biomarker can be problematic because the amount of blood protein in the blood depends on both the rate at which the muscle releases the protein and how quickly it is being cleared from the blood. In addition, blood CK activity has high intersubject variability, which does not seem to be related to sex, muscle mass, or activity level [1]. Not surprisingly, there was not a good relationship between the blood CK activity level and muscle damage after eccentric exercise [1]. It is interesting to note that the blood CK activity level did not change significantly after either bout of the eccentric exercise in the current study, even though most of the other EIMD markers suggested that there was muscle damage. It is even more interesting that CK seemed to decrease from pre- to post exercise in Bout 1 (Figure 6), although no statistically significant difference was detected. It is unclear as to why the CK results from the current study are inconsistent with those of previous studies [8,9,19], but it is probably due to the relatively higher baseline CK values of some of the subjects, the reasons for which are unknown. Regardless of the possible reasons, this seemingly unreasonable CK response after the first bout (which should have been novel to the subjects) suggests that the exercise was submaximal.

There are several limitations to the current study, and thus caution needs to be taken when generalizing the findings observed in this study. Firstly, we did not control for the menstrual cycle of the subjects. The midluteal phase has a higher estrogen level than prefollicular and midfollicular phases. Estrogen has been shown to provide a protective effect on muscle damage following eccentric exercise [42]. Thus, the different menstrual phases under which the two exercise bouts were performed might be a confounding factor in the current study. To compensate for that, we estimated each subject’s menstrual phase during both eccentric exercise bouts based on the first day of a subject’s menstrual bleeding, assuming the subjects had a 28-day menstrual cycle. Out of the 12 subjects whose data were included for most of the measured outcomes in the current study, eight subjects were in the follicular phase and three were in the luteal phase during Bout 1. One subject was on birth control medication and thus had no menstruation. We performed a two-way (time–menstrual cycle) repeated measures ANOVA to compare the isokinetic muscle strength in Bout 1 between the subjects in follicular phase vs. luteal phase and did not find any difference of statistical significance (data not shown), suggesting that the subjects with different menstrual phases had a similar muscle damage response after eccentric exercise in the current study. However, the menstrual phases in this study were roughly estimated and might not be accurate. For future studies on the CL-RBE in women, it is recommended to control for menstrual cycle and to verify the menstrual phases via measuring blood estrogen levels, basal body temperature, body water content, etc. Secondly, we recognized that the peak strength loss occurred immediately following the eccentric exercise, which could be due to the combining effects of muscle fatigue and damage instead of muscle damage alone. Consequently, it was not certain what the actual contribution of muscle damage is to the observed peak isokinetic muscle force deficits immediately post-exercise. Thirdly, without the EMG values collected from the maximal isometric contraction, we were not able to examine and compare the normalized EMG vales. This limited our ability to directly compare the muscle activation levels between the two bouts. Fourthly, we did not collect data on peak eccentric torque, rate of force decrement, or the angle at which peak torque was achieved during both eccentric exercise bouts; thus, we were unable to prove that the subjects had provided maximal efforts during the eccentric exercise. In fact, the values of the total work during the eccentric exercise in this study (400–500 J) were extremely low for 45 repetitions compared to values (5000–6000 J) from a previous study [43] in which healthy male subjects completed 60 maximal eccentric actions of the elbow flexors. Although the subjects were supposed to provide maximal effort during the eccentric contractions, these data suggested that eccentric exercise was actually performed at a submaximal level. Fifthly, the sample size used in this study was not sufficient as demonstrated by the observed small effects. Investigators seeking to apply these findings should do so with caution and consider these results as pilot data. Finally, two subjects stated that they felt as though the hand grip was putting more strain on their wrists compared to their biceps. This was unexpected because previous studies used the same position of the attachment. To minimize or completely avoid strain on the wrist, an attachment should be found so that the wrist of the subject will be strapped, and the subject will be able to relax her hand while doing eccentric contractions of their elbow flexors.

## 5. Conclusions

The CL-RBE of the elbow flexors was not evident in young healthy women in this study. This was probably because the mild muscle damage induced by the initial bout of the eccentric exercise was not severe enough to generate protective adaptation in the contralateral arm during the second bout or the CL-RBE in women lasted shorter than two weeks. Although the current study did not find the expected CL-RBE in women, it can be considered a pilot study and provides important information and guidance for warranted future studies on CL-RBE in women. For instance, future studies investigating the CL-RBE in women should consider using either more damaging eccentric exercise protocols such as exercise with more eccentric repetitions, higher intensity, or with shorter time intervals between the two exercise bouts. In addition, more studies are needed to uncover the mechanisms of the CL-RBE.

## Figures and Tables

**Table 1:** Baseline and pre-exercise values of isokinetic strength, ROM, and limb girth of the elbow flexors.

	Baseline	Pre-Exercise
Isokinetic peak torque (N·m)		
Bout 1	19.5 ± 4.5	19.4 ± 4.5
Bout 2	19.5 ± 5.9	17.6 ± 4.7
ROM (°)		
Bout 1	144.1 ± 5.3	145.8 ± 5.9
Bout 2	145.6 ± 4.7	145.1 ± 4.5
Limb girth (cm)		
Bout 1	28.6 ± 3.2	28.6 ± 3.1
Bout 2	28.7 ± 3.2	28.5 ± 3.2

Data are mean ± SD (*n* = 12).

## Data Availability

The data presented in this study are available on email request from the corresponding author.
